# Subhepatic Appendicitis Presenting With Recurrent Abdominal Pain

**DOI:** 10.7759/cureus.32939

**Published:** 2022-12-25

**Authors:** Adham H Yousef, Vugar Suleimanov

**Affiliations:** 1 General Surgery, Jubail General Hospital, Jubail, SAU; 2 Surgery, Jubail General Hospital, Jubail, SAU

**Keywords:** laparotomy, gut malrotation, cecum, acute appendicitis, subhepatic

## Abstract

Gut malrotation may result in failure of descent of the cecum to the right iliac fossa, resulting in the anomaly where the cecum and appendix are situated in the subhepatic/gallbladder region. Although the true incidence of subhepatic cecum or appendix is not known, there is a handful of case reports in the literature describing the diagnosis and management of subhepatic appendicitis and associated challenges. Some case reports describe subhepatic appendicitis, where the cecum is in a normal position, but the subhepatic tip of the long appendix gets perforated or inflamed, resulting in the process being in the subhepatic region. We report a patient with subhepatic appendicitis, who had multiple episodes of abdominal pain for almost one year, treated with antibiotics, but was never diagnosed properly. The case was diagnosed by abdominal sonography and was managed successfully in our institution.

## Introduction

Subhepatic appendicitis is a very uncommon entity, accounting for only 0.08 % of all cases of acute appendicitis according to a study of 7210 patients by Palanivelu et al. in 2007 [1]. The first case of subhepatic appendicitis was reported in 1955 by King; according to the report, the first description of the subhepatic appendix and cecum was done by professor Turner in 1863 at autopsy [2].

Acute appendicitis is one of the commonest causes of acute abdomen in children and young adults and there are countless studies in the literature on the subject. Although diagnosis and management of acute appendicitis are often straightforward, not infrequently surgeons face dilemmas in both diagnosis and management, due to variations in the anatomic position of the appendix [3]. Subhepatic appendicitis often mimics liver and biliary tract disease, which frequently ends up with a delay in diagnosis and unnecessary cholecystectomy, repeat admissions, increased morbidity, and cost [4].

Diagnosis of subhepatic appendicitis is usually made by contrast-enhanced computed tomography (CECT) of the abdomen, or upon diagnostic laparoscopy. Abdominal sonography has poor sensitivity for diagnosing unusually located appendicitis, especially in retrocecal cases [5]. We present a case of subhepatic acute appendicitis diagnosed by abdominal sonography only and timely surgical intervention precluded perforation, which is a common sequela of the subhepatic location of an inflamed appendix.

## Case presentation

**Figure 1:**
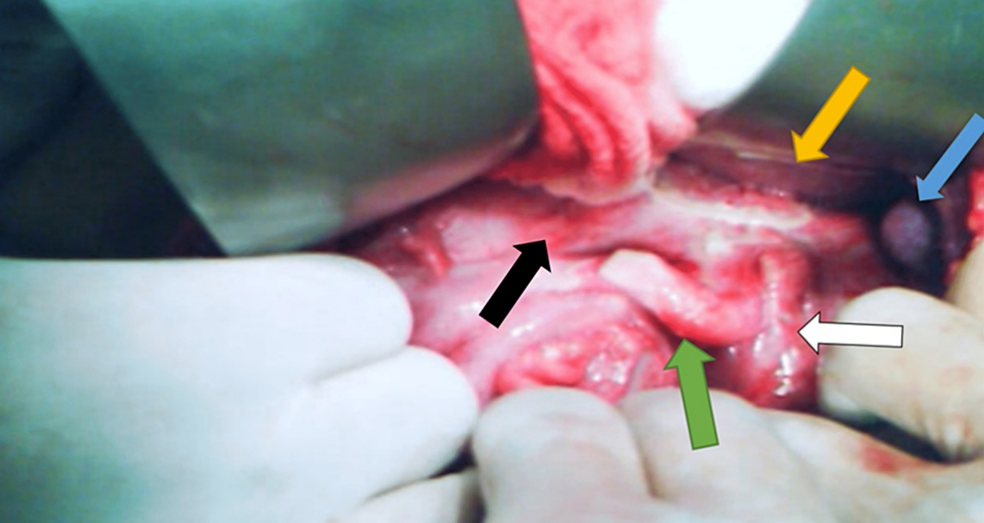
Intraoperative image showing inflamed subhepatic appendix (Green arrow), liver (Yellow arrow), gall bladder (Blue arrow), cecum (Black arrow), and ileum (White arrow).

A 25-year-old male patient presented to the emergency department (ED) of our district general hospital with a one-day history of right-sided abdominal pain, fever, nausea, anorexia, and vomiting. The patient admitted to having repeated similar attacks for the last nine months, which were managed by antibiotics with an improvement of the condition, only to recur after some time. He did not have abdominal sonography or computed tomography done for recurrent abdominal pain prior to presentation to our hospital. The patient had no surgery in the past and was not suffering from any chronic illness. On examination, he was febrile with a core body temperature of 38,2 ° C, but his remaining vital signs were within normal range. Abdominal examination revealed right-sided abdominal tenderness, which was more pronounced over the right upper quadrant with muscle guarding and positive rebound tenderness. His laboratory blood work showed an elevated leukocyte count of 15.0x10^9/L, with significant left shift, neutrophilia being 89%. Renal and liver function tests were normal. Radiographs of the chest and abdomen were unremarkable, but abdominal sonography showed evidence of appendicitis, with the liver and the biliary tree being normal. The sonographer reported that the inflamed appendix appears to be just under the gallbladder. Since laparoscopic set-up was not available in the hospital, open surgery was the only option for the management of our patient. The patient was taken to the operating room (OR) after antibiotic and thromboembolism prophylaxis, the abdomen was entered through the right pararectal incision in order to have good access to the subhepatic region. Upon exploration, we found the inflamed appendix and cecum in the subhepatic position (Figure 1).

The appendectomy was carried out smoothly since there was no perforation or mass formation. The patient had an uneventful recovery and was discharged home in good condition after two days.

## Discussion

An inflamed appendix is one of the most common causes of surgical abdomen in children and young adults. However, sometimes the diagnosis and management can prove challenging even to experienced clinicians due to the atypical presentation dictated by varying locations of the appendix. The retrocecal position of the appendix is reported to be the commonest location (74%), followed by the pelvic (21%) [6]. Some rare locations are reported too, like subhepatic, inside the hernia sac, mesocolic, in left iliac fossa, and lumbar [6]. If the appendix is in an atypical position, the patient’s presentation does not allow easy and straightforward diagnosis, leading to delay of management, perforation, abscess formation, and peritonitis, and may even result in a fatal outcome. Just like in our case, many patients with atypically situated appendixes get recurrent attacks of abdominal pain and receive unnecessary treatments before being diagnosed [6,7]. Subhepatic appendicitis is frequently mistaken for acute or chronic cholecystitis, and even some patients undergo laparoscopic or open cholecystectomy on an assumption of gall bladder disease, especially if concomitant gall stones are present [7,8].

After the first report of acute subhepatic appendicitis by King in 1955 [2], many similar reports describing the challenges of subhepatic appendicitis have appeared in the literature; most cases in adults, and some in children [9,10]. Being a cheap, readily available, and harmless (no ionizing radiation is involved) imaging modality with acceptable diagnostic yield for abdominal pathologies, ultrasonography has been used as the first option for most cases of acute abdomen [9]. However, in the case of subhepatic appendicitis, abdominal ultrasonography has been proven to be inferior compared to CECT [11]. Since our case was readily diagnosed by abdominal sonography, we did not contemplate using a CECT study, which entails additional cost and radiation.

Once the diagnosis of subhepatic appendicitis has been made, the optimal management approach must be contemplated, since there is no standard approach or internationally accepted consensus, considering the rare nature of the condition. It appears that the laparoscopic approach is preferred by most surgeons, which is subject to the availability of skilled surgeons and laparoscopic set-up, especially in the developing world [12,13]. Although there are multiple systematic reviews and some meta-analyses in the literature, proving the feasibility, safety, and advantages of laparoscopic appendectomy for normally situated appendixes [14-17], the experience of a minimally invasive approach to the subhepatic appendix is limited to a few case reports. The added advantage of a minimally invasive approach is that it gives access to any region of the abdomen without using long incisions, which increases morbidity significantly. Having said this, we must admit that the minimally invasive surgery (MIS) set-up and expertise of the surgeon in MIS are subject to availability, especially in developing countries, as mentioned earlier. That's why the operating surgeon must decide the best approach to the management of cases like this, based on the availability of laparoscopic set-up and advanced laparoscopic skills. We chose exploratory laparotomy because we did not have laparoscopic surgery equipment in our hospital.

## Conclusions

Subhepatic appendicitis is a rare condition, which poses a significant challenge to clinicians in terms of timely diagnosis and optimal management. The great majority of the cases of subhepatic appendicitis either get perforation with abscess, peritonitis, sepsis, and increased morbidity, or suffer from repeated attacks of abdominal pain before getting an accurate diagnosis. We recommend using abdominal sonography initially, which can be helpful in a good number of cases, but maintaining a lower threshold for CRCT for equivocal cases. Diagnostic laparoscopy, coupled with laparoscopic appendectomy, is a very useful tool in expert hands for both diagnosis and safe management of this rare and challenging condition.

## References

[1] Palanivelu C, Rangarajan M, John SJ, Senthilkumar R, Madhankumar MV (2007). Laparoscopic appendectomy for appendicitis in uncommon situations: the advantages of a tailored approach. Singapore Med J.

[2] King A (1955). Subhepatic appendicitis. AMA Arch Surg.

[3] Hakim M, Mostafa R, Al Shehri M, Sharawy S (2020). Surgical management of subhepatic perforated appendicitis: a case report. J Med Case Rep.

[4] Chiapponi C, Jannasch O, Petersen M, Lessel W, Bruns C, Meyer F (2017). A rare case of perforated "sub-hepatic appendicitis" - a challenging differential diagnosis of acute abdomen based on the combination of appendicitis and maldescent of the caecum. Pathol Res Pract.

[5] McAninch SA, Essenburg A (2019). Pediatric subhepatic appendicitis with elevated lipase. Am J Emerg Med.

[6] SI S, JH AK, SH N, MI TS (2014). A case of right upper abdominal pain misdiagnosed on computerized tomography. Malays J Med Sci.

[7] Ball WR, Privitera A (2013). Subhepatic appendicitis: a diagnostic dilemma. BMJ Case Rep.

[8] Kulvatunyou N, Schein M (2001). Perforated subhepatic appendicitis in the laparoscopic era. Surg Endosc.

[9] Alqahtani SM, Lasheen M, Paray S (2019). Subhepatic appendicitis in an 11-year-old boy: a case report. Cureus.

[10] Biçer Ş, Çelik A (2015). Duodenal obstruction caused by acute appendicitis with intestinal malrotation in a child. Am J Case Rep.

[11] Chong HC, Chai FY, Balakrishnan D, Asilah SM, Adila IN, Syibrah KZ (2016). Malrotated subhepatic caecum with subhepatic appendicitis: diagnosis and management. Case Rep Surg.

[12] Aneiros Castro B, Cano Novillo I, García Vázquez A, Yuste García P, Ferrero Herrero E, Gómez Fraile A (2018). Is the laparoscopic approach appropriate for pediatric subhepatic appendicitis?. Asian J Endosc Surg.

[13] Longani SK, Ahmed A (2019). Classical presentation of acute appendicitis in the case of a subhepatic appendix. Cureus.

[14] Aziz O, Athanasiou T, Tekkis PP (2006). Laparoscopic versus open appendectomy in children: a meta-analysis. Ann Surg.

[15] Golub R, Siddiqui F, Pohl D (1998). Laparoscopic versus open appendectomy: a metaanalysis. J Am Coll Surg.

[16] Markides G, Subar D, Riyad K (2010). Laparoscopic versus open appendectomy in adults with complicated appendicitis: systematic review and meta-analysis. World J Surg.

[17] Athanasiou C, Lockwood S, Markides GA (2017). Systematic review and meta-analysis of laparoscopic versus open appendicectomy in adults with complicated appendicitis: an update of the literature. World J Surg.

